# Effector-Memory γδ T Lymphocytes Predict CMV Disease After the Withdrawal of Prophylaxis in Kidney Transplant Recipients

**DOI:** 10.3389/ti.2025.14339

**Published:** 2025-07-16

**Authors:** Yoann Abadie, Jonathan Visentin, Elodie Wojciechowski, Manon Charrier, Julie Déchanet-Merville, Isabelle Garrigue, Patrick Blanco, Pierre Merville, Hannah Kaminski, Lionel Couzi

**Affiliations:** ^1^ Department of Nephrology, Transplantation, Dialysis and Apheresis, Bordeaux University Hospital, Bordeaux, France; ^2^ Centre National de la Recherche Scientifique - Unité Mixte de recherche (CNRS-UMR) 5164 ImmunoConcEpT, Bordeaux University, Bordeaux, France; ^3^ Laboratory of Immunology and Immunogenetics, Bordeaux University Hospital, Bordeaux, France; ^4^ Laboratory of Virology, Bordeaux University Hospital, Bordeaux, France

**Keywords:** CMV, infection, immunology, immunomonitoring, prophylaxis

## Abstract

Evaluation of CMV-specific cell-mediated immunity (CMI) has improved strategies to prevent post-transplant CMV disease. This study assessed the association between CMV disease and absolute count of TEMRA γδ T cells at the end of universal prophylaxis in kidney transplant recipients (KTR). We retrospectively analyzed 262 R⁺ and 82 D⁺/R⁻ KTRs who received antiviral prophylaxis and had TEMRA γδ T cells quantified at the end of prophylaxis. The primary endpoint was CMV disease within two years post-transplant. Post-prophylaxis CMV disease occurred in 43/344 (12.5%) patients. A threshold of 4.65/mm³ for TEMRA γδ T-cell count was identified by ROC analysis; higher counts were associated with reduced CMV disease incidence. While no significant association was found in the overall cohort, in R⁺ patients, a count >4.65/mm³ was associated with a 97.7% positive predictive value for protection against CMV disease. Multivariate analysis confirmed its independent association with disease-free survival [HR: 0.27 (95% CI: 0.09–0.85), p = 0.0252]. Measuring TEMRA γδ T-cell counts at the end of prophylaxis may serve as a useful, accessible immune marker to guide CMV prevention strategies in R⁺ kidney transplant recipients.

## Graphical Abstract

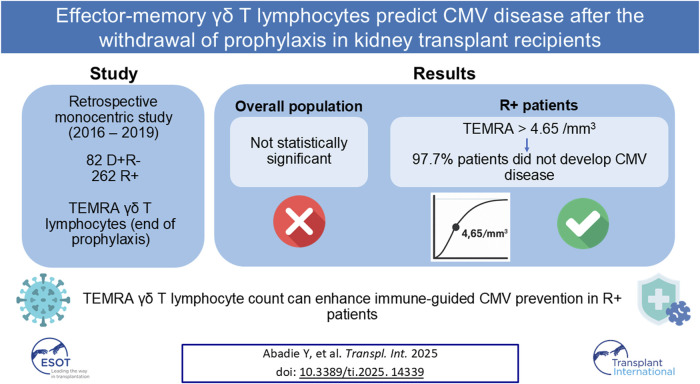

## Introduction

Human cytomegalovirus (CMV) is a widespread virus within the general population [1]. Although the infection is mostly asymptomatic in immunocompetent hosts, it can have severe consequences for immunocompromised patients. In particular, kidney transplant recipients (KTR) are at risk, as CMV can cause direct, life-threatening organ damage (e.g., colitis, pneumonitis, encephalitis) [2], and contribute to indirect complications such as acute rejection [3] or post-transplant diabetes mellitus [4]. These complications significantly reduce both patient and graft survivals [5]. Fortunately, substantial progress has been made in preventing CMV disease with the advent of the universal prophylaxis [6, 7].

A better understanding of the anti-CMV immune response has enabled the development of biomarkers that can stratify the risk of developing CMV disease. The most commonly used biomarker is based on donor and recipient serology, with donor-positive/recipient-negative (D+R-) patients being at the highest risk for CMV infection [3]. In recent years, additional biomarkers have been identified, focusing on the cellular component of the anti-CMV immune response, particularly the αβ T-cell response [8]. Two commercially available assays have been tested in various contexts. The QuantiFERON assay has demonstrated its value in 1) predicting protection against CMV disease in D+R- patients when performed at the end of prophylaxis [9, 10], 2) predicting spontaneous viral clearance in patients with low DNAemia [11], and 3) forecasting protection against clinical recurrence at the end of CMV treatment [12]. Similarly, ELISpot has proven effective in identifying KTR at very low risk of developing CMV disease [13–15].

Importantly, recent randomized trials have incorporated QuantiFERON or ELISpot to assess infection risk and personalize post-transplant CMV prevention strategies. Two trials confirmed the safety of discontinuing antiviral prophylaxis after 4–6 weeks in R+ patients who had received thymoglobulin, provided their QuantiFERON or ELISpot tests were positive, without increasing CMV infection rates [16, 17]. Moreover, Jarque et al. showed that R+ patients receiving basiliximab who had a positive ELISpot test 2 weeks post-transplant were protected from CMV infection [18].

Interestingly, some studies have shown that certain patients did not develop CMV disease despite the absence of any detectable CMV-specific αβ T-cell response, while others developed CMV disease despite having a CMV-specific αβ T-cell response [10]. These assays exclusively assess the αβ T-cell response, leading to the hypothesis that other components of the anti-CMV immune response may be essential to control the infection.

Our group has shown that the T cell immune response to CMV is also mediated by another subset of non-αβ T cells, namely the γδ T cells (and more specifically those negative for the Vδ2 TCR chain). The expansion of these cells during CMV infection correlates with the resolution of the viremia and the absence of recurrence [19]. *In vitro*, γδ T cells clones or cell lines have been shown to inhibit CMV replication and to kill CMV-infected cells [20]. This protective role has been confirmed by several mouse studies [21–23]. The expansion of the γδ T lymphocyte subset during CMV infection is accompanied by a very specific phenotypic change, including the acquisition of markers indicative of cytotoxic activity (perforin+, granzyme+) and of terminal effector differentiation characterized by the loss of CD27 and presence of CD45RA expression, (CD27^−^, CD45RA+) [24, 25] so called T effector/memory expressing CD45RA (TEMRA) phenotype.

In this study, we aimed to analyze the occurrence of CMV disease in relation to the absolute count of TEMRA γδ T cells at the end of universal prophylaxis in KTR.

## Materials and Methods

### Study Design and Population

We conducted this retrospective study at Bordeaux University Hospital (France). KTRs who received a deceased or living donor kidney between 1 September 2016 and 31 December 2019 were included if they were over 18 years old and if their CMV status was either D+R- or R+.

Induction therapy consisted of thymoglobulin for HLA-sensitized KTRs, and basiliximab for the others. Maintenance treatment included tacrolimus, targeting through level target of 8–10 ng/mL during the first year, followed by 6–10 ng/mL, along with mycophenolic acid (720 mg bid). Steroids were rapidly reduced to 5 mg/day and weaned in non-HLA-sensitized KTRs during the first month post-transplantation. Everolimus was used for a small number of KTRs with a through level target of 5–8 ng/mL.

All KTRs received universal prophylaxis with valganciclovir, aiming for 6 months in D+R- KTRs or 3 months in R+ KTRs. Valganciclovir dosage adjustments were made using the Cockcroft-Gault formula.

KTRs were excluded if they did not take antiviral prophylaxis for at least 6 weeks, if they experienced death, graft loss, or were lost-of follow-up before month 3, and if monitoring of the γδ T lymphocyte subset was not performed at the end of the antiviral prophylaxis. Notably, γδ T lymphocyte measurement at the end of the prophylaxis was part of the routine monitoring of KTRs during this period.

All clinical and biological variables were collected from the R@N database (with final approval from the French Data Protection Authority [CNIL], number 135715). All participants gave written informed consent. The study was performed in accordance with the ethical standards as laid down in the Declaration of Helsinki, and was approved by the Institutional Review Board of the Bordeaux University Hospital.

### Endpoints

The endpoints were:
1) The incidence of CMV disease during the first 2 years post-transplantation, based on the absolute count of lymphocytes, Vδ2^neg^ γδ T lymphocytes, and TEMRA Vδ2^neg^ γδ T lymphocytes measured at the end of universal prophylaxis in the overall population.
2) The incidence of CMV disease in R+ KTRs, according to the same lymphocyte and T cell subsets counts.

### Definitions

CMV disease was defined as “CMV syndrome” or “probable or proven end-organ CMV disease” using standardized criteria from international guidelines [26].

CMV syndrome was defined by the detection of a positive CMV PCR, with at least 2 additional criteria among the following: fever, malaise or fatigue, leukopenia or neutropenia, thrombopenia or elevation of hepatic aminotransferase.

Proven CMV end-organ disease was defined as the presence of appropriate clinical symptoms together with documentation of CMV in tissue from the relevant organ by immunohistochemistry.

Probable CMV end-organ disease was defined as the presence of appropriate clinical symptoms together with documentation of high viral DNA levels in tissue from the relevant organ by quantitative nucleic acid testing.

The onset of CMV disease was marked by the first detection of CMV DNAemia with CMV symptoms. The duration of CMV disease was the time from the first positive CMV DNAemia until symptom resolution and viral eradication following at least 2 weeks of treatment. The treatment duration was defined as the period during which KTRs received antiviral therapy for CMV disease. Recurrent disease referred to a new episode in KTRs who had previously achieved negative CMV DNAemia following treatment.

### CMV Quantitative Nucleic Acid Testing

Various CMV quantitative nucleic acid testing (QNAT) methods were used throughout the study. Starting in September 2016, QNAT was performed with the LightMix^®^ Human Cytomegalovirus Kit (TIB MOLBIOL GmbH, Berlin, Germany), with detection and quantification thresholds of 250 and 1000 IU/mL, respectively. From April 2019 onward, the CMV R-GENE^®^ Kit (Biomerieux, France) was used, with thresholds of 150 and 200 IU/mL. All QNAT assays were conducted in the Department of Virology at Bordeaux University Hospital, adhering strictly to Quality Control for Molecular Diagnostics (QCMD, Glasgow, Scotland) standards since 2004. A CMV QNAT result below the quantification limit was considered negative.

### Flow Cytometry Analysis of Vδ2^neg^ γδ T Cells at the End of the Prophylaxis

Lymphocyte and Vδ2^neg^ γδ T lymphocyte counts were analyzed at the end of universal valganciclovir prophylaxis (±1 month). Vδ2^neg^ γδ T lymphocyte counts were determined by flow cytometry in the Department of Immunology and Immunogenetics at Bordeaux University Hospital, as previously described [19]. To identify the Vδ2^neg^ γδ T lymphocyte subset and their TEMRA phenotype, we used a panel containing antibodies targeting CD3, γδ TCR, Vδ2 TCR, CD27, and CD45RA (Beckman Coulter, Marseille, France). The Vδ2^neg^ γδ T lymphocyte subset is rare in CMV-naïve subjects [24]. Results were reported as “not interpretable” (NI) when fewer than 300 events were detected in the Vδ2^neg^ γδ T lymphocyte gate (Supplementary Figure S1). For clarity, TEMRA Vδ2^neg^ γδ T cells are referred to as TEMRA γδ T cells throughout this report.

### Other Variables Assessment

When comparing the incidence of CMV disease across different medication regimens or rejection episodes, only events occurring before the CMV disease onset were included in the “CMV disease” group. All rejection episodes were biopsy-proven. Preformed donor-specific antibodies (DSA) were defined as those present on the day of transplantation or earlier. Post-transplant estimated glomerular filtration rate (eGFR) was defined as the highest eGFR recorded during the prophylaxis period.

### Statistical Analysis

KTRs characteristics are presented as medians and interquartile ranges (IQR) for quantitative variables and as percentages for qualitative variables. Fisher’s exact test or McNemar’s test was used to compare qualitative variables, while Student’s t-test or the Mann–Whitney test was applied to quantitative variables. A p-value <0.05 was considered statistically significant. The relationship between Vδ2^neg^ γδ T lymphocyte counts was assessed using Spearman’s correlation (rho). Receiver operating characteristic (ROC) curve analysis was conducted to evaluate the performance of lymphocyte counts and TEMRA Vδ2^neg^ γδ T lymphocyte counts in predicting protection against CMV disease. The probability of CMV disease-free survival, based on lymphocyte levels, was estimated using the Kaplan-Meier method, and the log-rank test was used to compare hazards of CMV disease. Univariate Cox regression analysis was initially applied to identify variables associated with CMV disease. No continuous variable deviated from the assumption of linearity. Covariates with p-values <0.25 in univariate analysis were included in multivariate Cox regression analysis, and variables with p-values <0.05 were retained. Results are presented as hazard ratios (HR) with 95% confidence intervals (95% CI). All analyses were performed using RStudio (version 1.1.423; RStudio Inc., Boston, MA, United States) and Prism (version 10.0.2; GraphPad Software, Boston, MA, United States).

## Results

### Study Population

**FIGURE 1:**
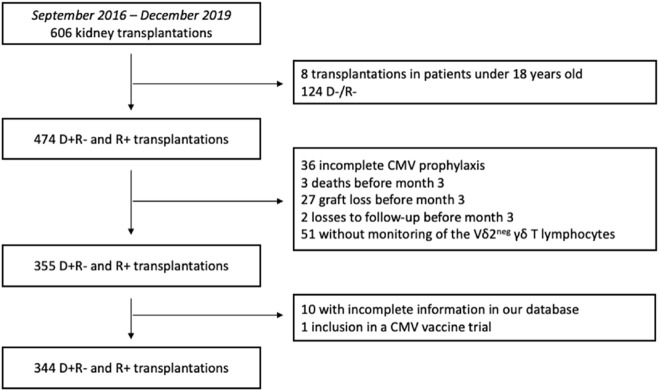
Flowchart of the study design. D+R-: Donor positive and recipient negative for CMV serology. D-R-: Donor negative and recipient negative for CMV serology. R+: Recipient positive for CMV serology.

Between September 2016 and December 2019, 606 kidney transplants were performed at Bordeaux University Hospital. Based on the inclusion and exclusion criteria, 344 KTRs were eligible for inclusion in the study (Figure 1).

Table 1 outlines the baseline characteristics of these patients. During the first 2 years post-transplantation, 43 out of 344 KTRs (12.5%) developed CMV disease, with a median onset of 79 days (IQR: 44.0–122.5 days) after discontinuing prophylaxis. Among these, 9 KTRs (20.9%) experienced CMV viral syndrome, and 34 KTRs (79.1%) developed CMV tissue-invasive disease. The median peak CMV DNAemia was 50,320 IU/mL (IQR: 12,432–281,010 IU/mL). The median disease duration was 29.5 days (IQR: 21.5–43 days), and the median treatment duration was 44 days (IQR: 24–55.5 days). CMV recurrence occurred in 6 of the 43 patients (13.6%).

**TABLE 1:** Baseline characteristics in the study population.

Characteristics	Total (N = 344)	No CMV disease (N = 301)	CMV disease (N = 43)	*p* value
Age, y, mean (SD)	56.5 (14.5)	56.8 (14.6)	54.2 (13.9)	0.22
Sex, M/F, No.	217/127	189/112	15/28	0.86
Previous kidney transplantation	74 (21.5%)	67 (22.2%)	7 (16.2%)	0.43
Serostatus				<0.01
D + R-	82 (23.8%)	56 (18.6%)	26 (60.4%)	
R+	262 (76.2%)	245 (81.4%)	17 (39.6%)	
Prophylaxis duration, d, median (IQR)				
D +R-	181 (134.8–183.0)	181 (146.5–183.0)	181.5 (98.75–183.3)	0.74
R+	91.5 (89.0–92.0)	91 (89.00–92.00)	92 (89.50–94.00)	0.63
Donor sex, M/F, n	183/155	159/136	24/19	0.87
Donor age, y, mean (SD)	58.5 (16.2)	58.5 (16.3)	58.0 (15.3)	0.97
Donor status				
Living donor	63 (18.4%)	53 (17.6%)	10 (23.6%)	0.40
Standard criteria donor	108 (31.3%)	98 (32.6%)	10 (23.3%)	0.29
Extended criteria donor	173 (50.3%)	150 (49.8%)	23 (53.5%)	0.74
Immunological risk				
No donor-specific antibodies	267 (77.6%)	232 (77.0%)	35 (81.3%)	0.69
Donor-specific antibodies	77 (22.4%)	69 (23.0%)	8 (18.7%)	0.69
Induction therapy				
No induction therapy	9 (2.7%)	8 (2.6%)	1 (2.3%)	>0.99
Basiliximab	153 (44.4%)	135 (44.8%)	18 (41.9%)	0.75
Thymoglobulin	183 (53.1%)	159 (52.8%)	24 (55.8%)	0.74
Maintenance therapy				
Tacrolimus	293 (85.2%)	253 (84.0%)	40 (93.0%)	0.16
Ciclosporin	51 (14.8%)	48 (16.0%)	3 (7%)	0.16
Steroid	295 (85.7%)	260 (86.4%)	35 (81.4%)	0.35
Mycophenolate	317 (92.1%)	276 (91.7%)	41 (95.3%)	0.55
Azathioprine	22 (6.4%)	19 (6.3%)	3 (7.0%)	0.74
mTOR inhibitors	65 (18.9%)	62 (20.6%)	3 (7.0%)	0.03
Antibody-mediated rejection	12 (3.5%)	11 (3.6%)	1 (2.3%)	>0.99
T-cell mediated rejection	37 (10.8%)	31 (10.3%)	6 (14.0%)	0.44
Time to rejection, d, median (IQR)	117 (70–383.3)	242 (77.5–386.8)	76.5 (28.25–103)	0.08
Ischemia time, mn, median (IQR)	747 (470.5–1,015)	749.5 (472.0–1,022)	729 (268.5–1,439)	0.36
Post-transplantation eGFR, mL/min/1.73m^2^, median (IQR)	35.0 (24.25–50.0)	35,0 (24.0–50.0)	39.0 (28.0–49.0)	0.48
2 years graft loss	13 (3.8%)	11 (3.7%)	2 (4.7%)	0.67
2 years death	17 (4.9%)	15 (5.0%)	2 (4.5%)	>0.99

SD: standard deviation.

M/F: Male/Female.

D+R-: Donor positive and recipient negative for CMV serology.

R+: Recipient positive for CMV serology.

IQR: interquartile range.

n: Number.

y: Year.

mn: Minutes.

mTOR: mammalian target of rapamycin.

CMV disease occurred in 31.7% (26/82) of D+R- KTRs and 6.4% (17/262) of R+ patients (p < 0.01). Conversely, CMV disease occurred in 4.6% (3/65) of patients treated with mTOR inhibitors and 14.3% (40/279) of patients not treated with mTOR inhibitors (*p* = 0.03). Interestingly, no significant differences were observed regarding the use of thymoglobulin or the number of treated acute rejection episodes between the groups.

CMV disease characteristics in D+R- and R+ subgroups are detailed in Supplementary Table S1.

### TEMRA γδ T Lymphocyte Count at the End of the Prophylaxis Is Higher in KTRs Without CMV Disease

We did not observe any episode of CMV disease before the γδ T lymphocyte measurement at the end of the prophylaxis.

Table 2A describes immune profiles of the “CMV disease” and “No CMV disease” KTRs. Vδ2^neg^ γδ T cells count was higher in the “No CMV disease” group than in the “CMV disease” group (18.4 ± 25.7/mm^3^ *versus* 6.0 ± 7.9/mm^3^; *p* < 0.01). TEMRA γδ T lymphocytes count was also higher in the “No CMV disease” group (23 ± 26.8/mm^3^ *versus* 4.6 ± 6.7/mm^3^; *p* < 0.01). Immune profiles in the D+R- and R+ subgroups are depicted in the Tables 2B, C.

**TABLE 2:** Immune characteristics at the end of the prophylaxis, overall and according to serotype.

A) Overall	No CMV disease, n = 301	CMV disease, n = 43	*p* value
Lymphocytes count, G/L, mean, SD	0.92 (0.58)	0.76 (0.49)	0.06
Vδ2^neg^ γδ T lymphocyte count,/mm^3^, mean, SD	18.4 (25.7)	6 (7.9)	<0.02
TEMRA γδ T lymphocyte count,/mm^3^, mean, SD	23 (26.8)	4.6 (6.7)	<0.01
TEMRA γδ T lymphocyte not interpretable, n, %	137 (45.5%)	24 (55.8%)	0.3

B) D + R-	No CMV disease, n = 56	CMV disease, n = 26	*p* value
Lymphocytes count, G/L, mean, SD	0.74 (0,42)	0.77 (0.54)	0.87
Vδ2^neg^ γδ T lymphocyte count,/mm^3^, mean, SD	8.93 (17.17)	12.05 (13.74)	0.14
TEMRA γδ T lymphocyte count,/mm^3^, mean, SD	13.52 (22.5)	14.31 (16.11)	0.57
TEMRA γδ T lymphocyte not interpretable, n, %	43 (76.8)	17 (65.4)	0.29

C) R+	No CMV disease, n = 245	CMV disease, n = 17	*p* value
Lymphocytes count, G/L, mean, SD	0.97 (0.60)	0.79 (0.42)	0.18
Vδ2^neg^ γδ T lymphocyte count,/mm^3^, mean, SD	22.51 (26.75)	10.56 (9.09)	0.04
TEMRA γδ T lymphocyte count,/mm^3^, mean, SD	24.98 (27.01)	8.28 (8.04)	<0.01
TEMRA γδ T lymphocyte not interpretable, n, %	93 (37.9%)	7 (41.1%)	0.87

D) Comparison of D + R- and R+ patients	D+ R- patients n = 82	R+ patients n = 262	*p* value
Lymphocytes count, G/L, mean, SD	0.74 (0.46)	0.95 (0.59)	<0.01
Vδ2^neg^ γδ T lymphocyte count,/mm^3^, mean, SD	9.9 (6.15)	21.72 (26.1)	<0.01
TEMRA γδ T lymphocyte count,/mm^3^, mean, SD	13.84 (19.72)	23.94 (26.54)	<0.01
TEMRA γδ T lymphocyte not interpretable, N, %	60 (73)	100 (38)	<0.01

SD: standard deviation.

n: Number.

D + R-: Donor positive and recipient negative for CMV, serology.

R+: Recipient positive for CMV, serology.

NI: not interpretable.

It is worth noting that a significant number of immunophenotyping assays did not yield interpretable TEMRA γδ T lymphocyte counts (161/344, 46.8%). These results, labeled as NI (not interpretable), were evenly distributed between the “CMV disease” and “No CMV disease” groups. The proportion of NI patients was similar between those who received thymoglobulin and those who did not (78/183 vs. 82/161, *p* = 0.13).

Patients with NI results had lower total lymphocytes counts and lower Vδ2^neg^ γδ T lymphocyte counts than patients with interpretable results, respectively 0.82 G/L vs. 1.01 G/L (*p* < 0.01) and 4.61/mm^3^ vs. 27.4/mm^3^ (*p* < 0.01) (Supplementary Table S2).

### TEMRA γδ T Lymphocytes Count >4.65/mm^3^ at the End of the Prophylaxis Is Associated With Protection Against CMV Disease

**FIGURE 2:**
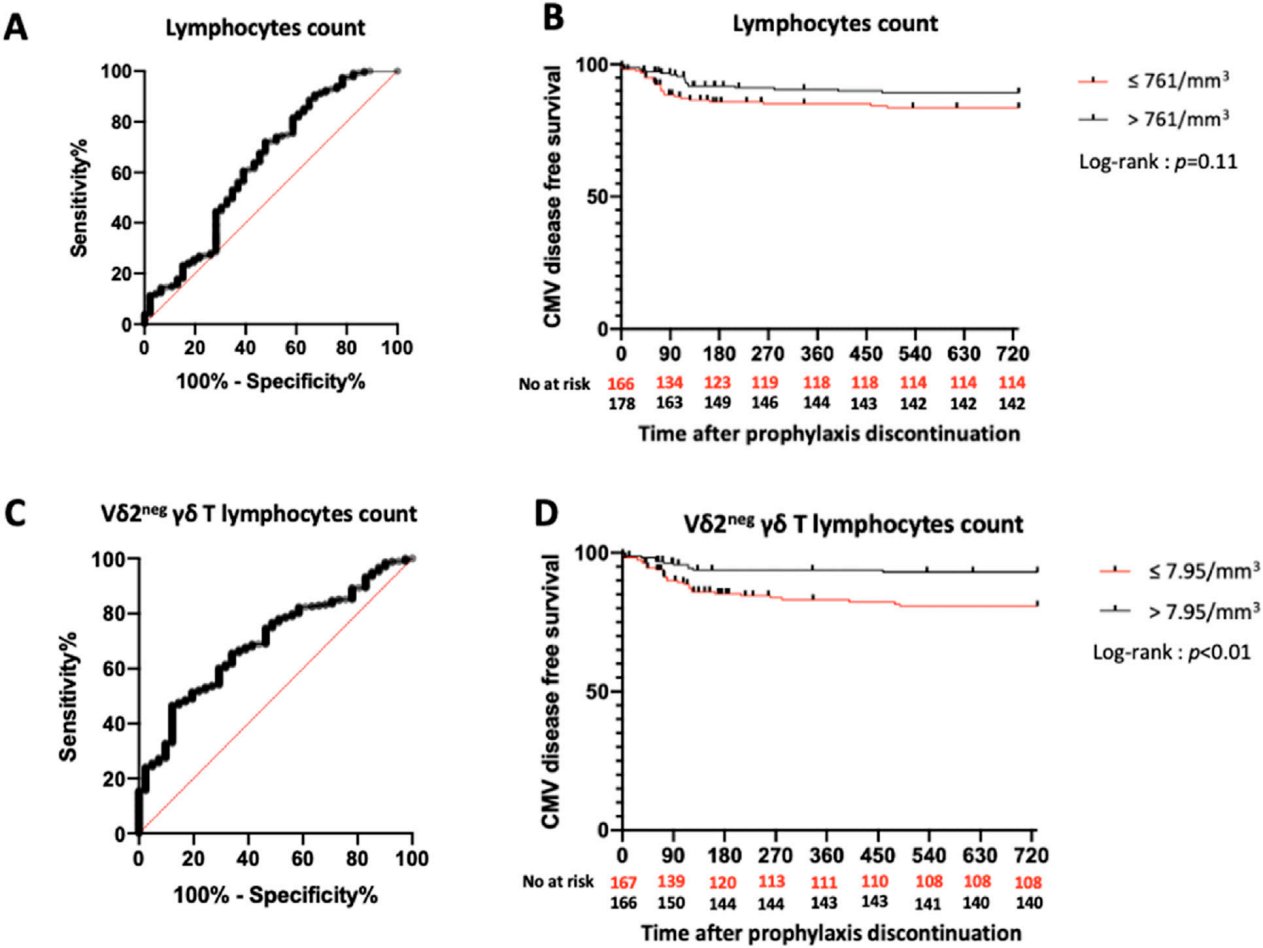
Predictive value of total lymphocytes and Vδ2^neg^ γδ T lymphocytes count in the overall study population. **(A)**: ROC curve of total lymphocytes count. AUC = 0.63. **(B)**: Incidence of CMV disease according to lymphocytes count. **(C)**: ROC curve of Vδ2^neg^ γδ T lymphocytes count. AUC = 0.70. **(D)**: Incidence of CMV disease according to Vδ2^neg^ γδ T lymphocytes count. ROC: Receiver Operating Characteristic. AUC: Area Under Curve.

ROC curve analyses for total lymphocyte and Vδ2^neg^ γδ T lymphocyte counts regarding CMV disease occurrence showed low AUCs of 0.63 and 0.70, respectively (Figures 2A,C). Given the low AUC, we assessed CMV disease-free survival by comparing patients with values above or below the median (lymphocyte count: 761/mm^3^; Vδ2^neg^ γδ T lymphocyte count: 7.95/mm^3^). The probability of CMV disease-free survival was similar between the “high lymphocyte” and “low lymphocyte” groups (*p* = 0.11) (Figure 2B). However, KTRs with a Vδ2^neg^ γδ T lymphocyte count >7.95/mm^3^ had a higher probability of CMV disease-free survival (*p* < 0.01) (Figure 2D).

**FIGURE 3:**
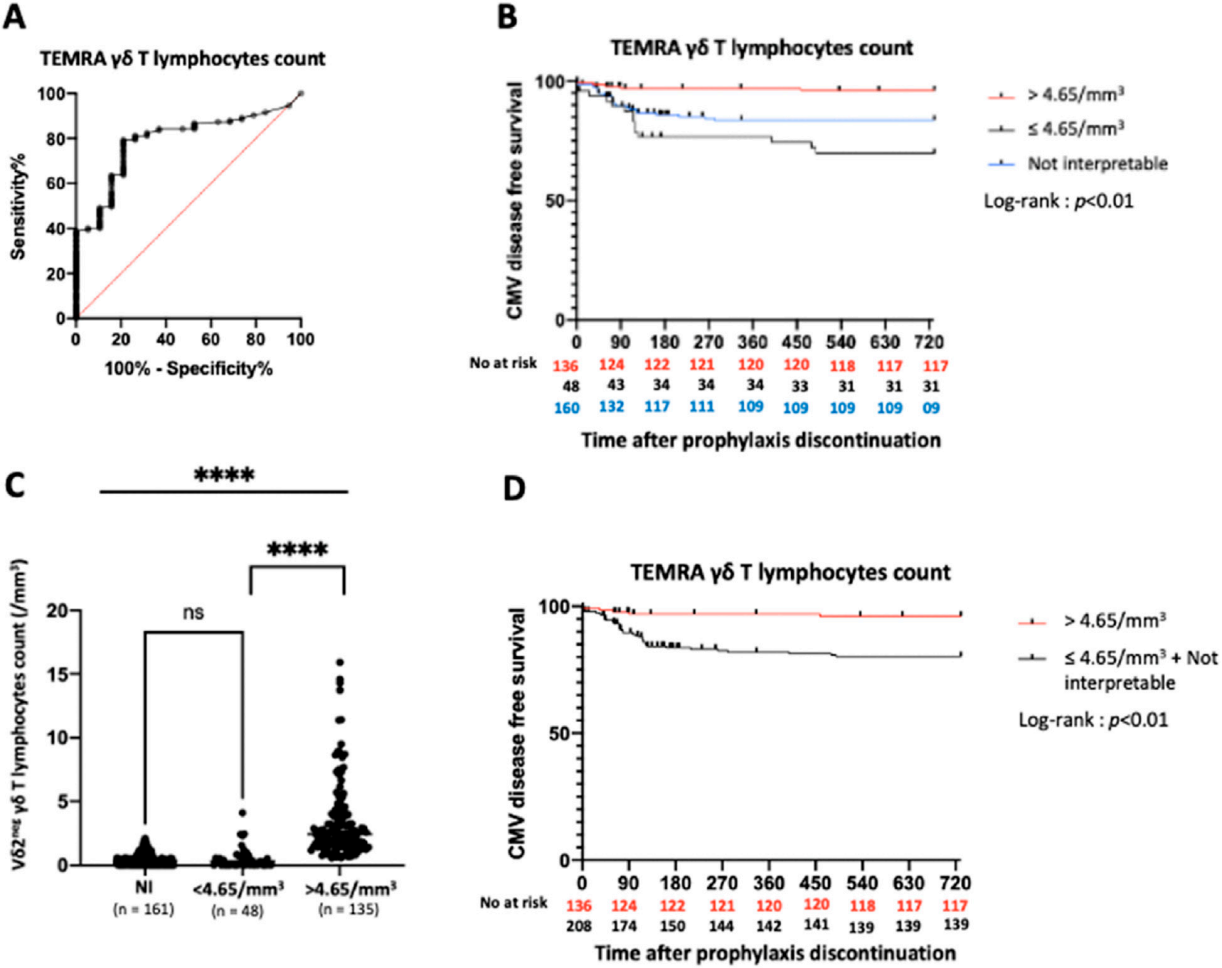
Predictive value of TEMRA γδ T lymphocytes count in the overall study population. **(A)**: ROC curve of TEMRA γδ T lymphocytes count. AUC = 0.79. **(B)**: Incidence of CMV disease according to TEMRA γδ T lymphocytes count (3 groups). **(C)**: Vδ2^neg^ γδ T lymphocytes count according to Vδ2neg γδ T lymphocytes percentage ****: *p* < 0.01. **(D)**: Incidence of CMV disease according to TEMRA γδ T lymphocytes count (2 groups). ROC: Receiver Operating Characteristic. AUC: Area Under Curve. NI: Not interpretable.

The ROC curve for TEMRA γδ T lymphocyte count (excluding NI KTRs) yielded an AUC of 0.79 and defined an optimal threshold of 4.65/mm^3^ (sensitivity 79.4%, specificity 78.9%) (Figure 3A). Of the 344 KTRs, 135 (39.2%) had a TEMRA γδ T lymphocyte count >4.65/mm^3^. KTRs with a count >4.65/mm^3^ had a higher probability of CMV disease-free survival compared to those with a count classified as NI or ≤4.65/mm^3^ *(p <* 0.01) (Figure 3B).

We further analyzed the 161 NI KTRs and found that their Vδ2^neg^ γδ T lymphocyte counts were very low, similar to those of KTRs with TEMRA γδ T lymphocyte counts ≤4.65/mm^3^, and much lower than those with counts >4.65/mm^3^ [median: 3.2/mm^3^ (IQR: 0.1–7.25), 2.9/mm^3^ (IQR: 0.2–8.15), 24.4/mm^3^ (IQR: 16.15–40.58), respectively] (Figure 3C). Thus, we grouped KTRs with TEMRA γδ T lymphocyte counts classified as NI and ≤4.65/mm^3^. KTRs with TEMRA γδ T lymphocyte counts >4.65/mm^3^ had significantly higher CMV disease-free survival rates than those in the combined NI and ≤4.65/mm^3^ group (*p* < 0.01) (Figure 3D).

We conducted a univariate analysis to identify factors associated with CMV disease (Table 3). The following factors were included in the multivariate analysis: R+ serostatus [HR 0.16 (95% CI 0.09–0.29); *p* < 0.01], total lymphocyte count >761/mm^3^ [HR 0.56 (95% CI 0.30–1.05); *p* = 0.07], Vδ2^neg^ γδ T lymphocyte count >7.95/mm^3^ [HR 0.34 (95% CI 0.17–0.68); *p* < 0.01], TEMRA γδ T lymphocyte count >4.65/mm^3^ [HR 0.18 (95% CI 0.07–0.46); *p* < 0.01], and the use of mTOR inhibitors [HR 0.27 (95% CI 0.06–1.11); *p* = 0.07]. In the multivariate analysis, only R+ serostatus [HR 0.23 (IQR 0.11–0.45); *p* < 0.01] remained independently associated with CMV disease. The TEMRA γδ T lymphocyte count was no longer significantly associated with CMV disease [HR 0.39 (95% CI 0.14–1.09); *p* = 0.07] (Table 4).

**TABLE 3:** Univariate analysis of CMV disease risk factors in the study population.

Variables	HR	95% CI	*p* value
Age	1	0.99–1.01	0.89
Sex (reference group: male)	1.05	0.56–1.97	0.87
Thymoglobulin	1.21	0.66–2.20	0.54
mTOR inhibitors before CMV disease	0.27	0.06–1.11	0.07
Steroids	0.96	0.49–1.91	0.91
Rejection before CMV disease	1.00	0.99–1.00	0.30
R+ patients	0.16	0.09–0.29	<0.01
Lymphocytes count >0.761 G/L	0.56	0.30–1.05	0.07
Vδ2^neg^ γδ T lymphocyte count >7.95/mm^3^	0.34	0.17–0.68	<0.01
TEMRA γδ T lymphocyte count >4.65/mm^3^	0.18	0.07–0.46	<0.01

HR: hazard ratio.

CI: confidence interval.

mTOR: mammalian target of rapamycin.

R+: Recipient positive for CMV, serology.

**TABLE 4:** Multivariate analysis of CMV disease risk factors in the study population.

Variables	HR	95% CI	*p* value
mTOR inhibitors before CMV disease	0.28	0.07–1.15	0.08
R+ patients	0.22	0.11–0.45	<0.01
TEMRA γδ T lymphocyte count >4.65/mm^3^ *versus* ≤4.65/mm^3^ and NI	0.39	0.14–1.09	0.07

HR: hazard ratio.

CI: confidence interval.

mTOR: mammalian target of rapamycin.

R+: Recipient positive for CMV, serology.

NI: not interpretable.

### Differences of γδ T Lymphocyte Response Between D+/R- and R+ Patients at the End of the Prophylaxis

Total lymphocyte counts and Vδ2^neg^ γδ T lymphocyte counts were significantly lower in D+R- patients compared to R+ patients (0.74 ± 0.46 G/L vs. 0.95 ± 0.59 G/L, *p* < 0.01; Vδ2^neg^ : 9.9 ± 6.15 vs. 21.72 ± 26.1; *p* < 0.01 (Table 2D).

**FIGURE 4:**
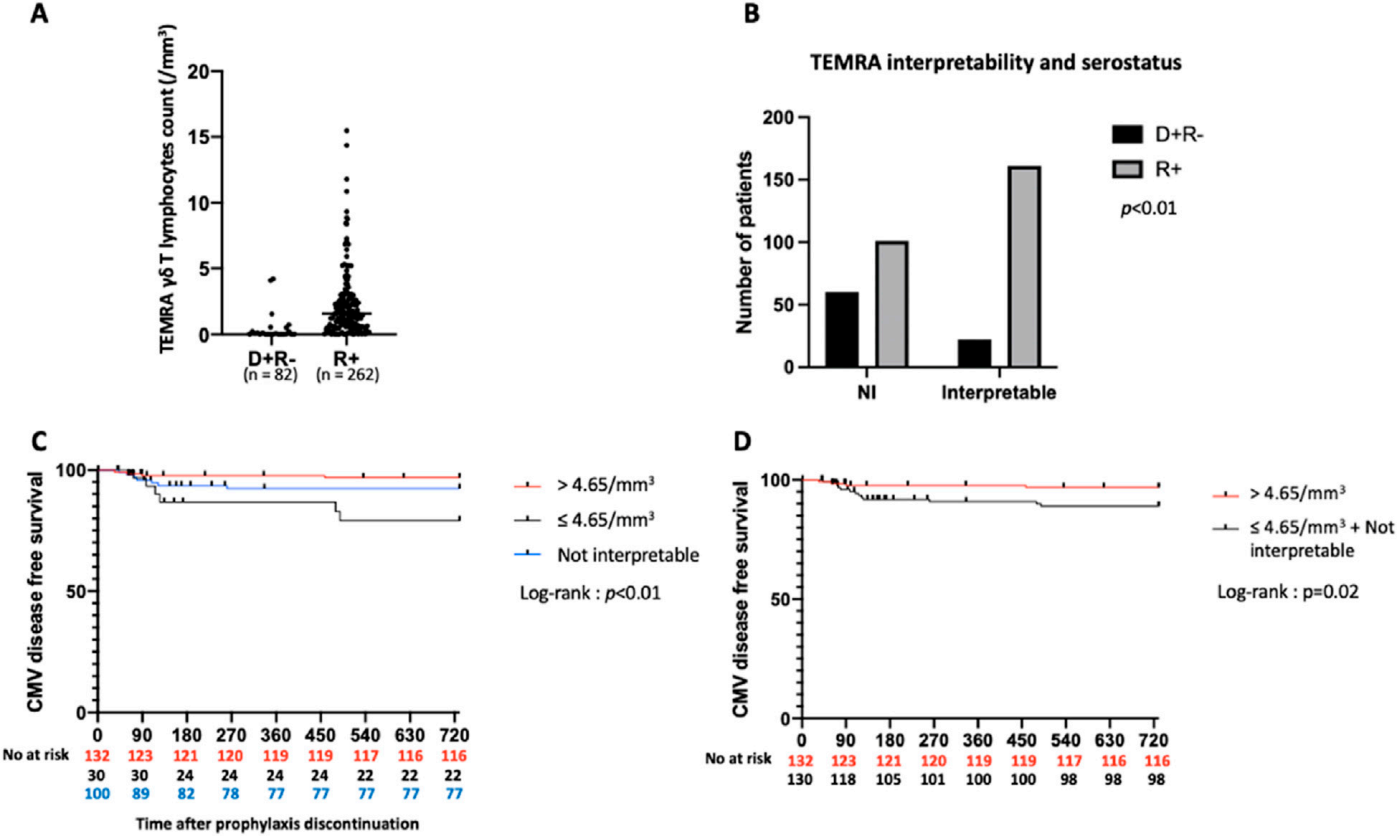
Predictive value of TEMRA γδ T lymphocytes count in R+ patients. **(A)**: TEMRA γδ T lymphocytes count according to serostatus. *p* < 0.01. **(B)**: TEMRA γδ T lymphocytes count interpretability according to serostatus. **(C)**: Incidence of CMV disease according to TEMRA γδ T lymphocytes count in R+ patients (3 groups). **(D)**: Incidence of CMV disease according to TEMRA γδ T lymphocytes count in R+ patients (2 groups). NI: Not interpretable. D+R-: Donor positive and recipient negative for CMV serology. R+: Recipient positive for CMV serology.

TEMRA : 13.84 ± 19.72 vs. 23.94 ± 26.54; *p* < 0.01 (Figure 4A). Among the 135 patients with TEMRA γδ T lymphocyte counts >4.65/mm^3^, only 4 were D+R-, the majority being R+ (n = 131) (*p* < 0.01). Finally, the number of interpretable results was lower in D+R- patients compared to R+ patients (22 *versus* 161) (Figure 4B).

Based on these findings, we evaluated the predictive value of a TEMRA γδ T lymphocyte count >4.65/mm^3^ for protection against CMV disease in the subgroup of R+ KTR, as detailed in Table 5.

**TABLE 5:** Baseline characteristics in the R+ population.

Characteristics	Total (N = 262)	No CMV disease (N = 245)	CMV disease (N = 17)	*p* value
Age, y, mean (SD)	57.1 (14.4)	57.1 (14.5)	57.6 (13.4)	0.94
Sex, M/F, No.	160/102 (61%/39%)	149/96 (61%/39%)	11/6 (64%/36%)	0.80
Previous kidney transplantation	62 (23.7%)	58 (23.7%)	4 (23.5%)	>0.99
Prophylaxis duration, d, median (IQR)	91.5 (89.0–92.0)	91 (89.00–92.00)	92 (89.50–94.00)	0.63
Donor sex, M/F, No	147/112	140/102	7/10	0.21
Donor age, y, mean (SD)	58.7 (12.6)	58.6 (15.7)	60.3 (14.5)	0.56
Donor status				
Living donor	44 (16.8%)	41 (16.7%)	3 (17.6%)	>0.99
Standard criteria donor	85 (32.4%)	82 (33.5%)	3 (17.6%)	0.28
Extended criteria donor	133 (50.8%)	122 (49.8%)	11 (64.8%)	0.31
Immunological risk				
No donor-specific antibodies	199 (76.0%)	191 (78.0%)	8 (47.0%)	<0.01
Donor-specific antibodies	63 (24.0%)	54 (22.0%)	9 (53.0%)	<0.01
Induction therapy				
No induction therapy	8 (3.0%)	7 (2.9%)	1 (5.9%)	0.41
Basiliximab	112 (42.7%)	107 (43.7%)	5 (29.4%)	0.31
Thymoglobulin	143 (54.6%)	132 (53.9%)	11 (64.7%)	0.45
Maintenance therapy				
Tacrolimus	228 (87.0%)	212 (86.5%)	16 (94.1%)	0.70
Ciclosporin	34 (13.0%)	33 (13.5%)	1 (5.9%)	0.70
Steroid	202 (77.0%)	187 (76.3%)	15 (88.2%)	0.37
Mycophenolate	241 (92.0%)	224 (91.4%)	17 (100%)	<0.01
Azathioprine	12 (4.5%)	12 (4.9%)	0 (0%)	>0.99
mTOR inhibitors	52 (19.8%)	51 (20.8%)	1 (5.9%)	0.20
Antibody-mediated rejection	10 (3.8%)	10 (4.1%)	0 (0.0%)	>0.99
T-cell mediated rejection	30 (11.5%)	25 (10.2%)	5 (29.4%)	0.03
Time to rejection, d, median (IQR)	116 (54–383)	117 (67–283)	62 (14–110)	0.26
Ischemia time, mn, median (IQR)	749.0 (495.5–1,013)	749.5 (491.3–1,015)	743 (395.0–1,004)	0.94
Post-transplantation eGFR, mL/min/1,73m^2^, median (IQR)	36.5 (24.75–50.25)	38.0 (25.0–51.5)	28.0 (23.5–34.0)	0.02
2 years graft loss	10 (3.8%)	9 (3.7%)	1 (5.9%)	0.49
2 years death	10 (3.8%)	9 (3.7%)	1 (5.9%)	0.49

SD: standard deviation.

M/F: Male/Female.

D + R-: Donor positive and recipient negative for CMV, serology.

R+: Recipient positive for CMV, serology.

IQR: interquartile range.

n: Number.

y: Year.

mn: Minutes.

mTOR: mammalian target of rapamycin.

### TEMRA γδ T Lymphocytes Count >4.65/mm^3^ at the End of the Prophylaxis Is Independently Associated With a Protection Against CMV Disease in R+ KTRs

Of the 262 R+ KTRs (including NI KTRs), 131 (50%) had a TEMRA γδ T lymphocyte count >4.65/mm^3^. The sensitivity of this test was low (52.2%), but specificity was high (82.3%). The positive predictive value (i.e., protection against CMV disease in KTRs with a TEMRA γδ T lymphocyte count >4.65/mm^3^) was 97.7%, while the negative predictive value was only 10.7%. R+ KTRs with a TEMRA γδ T lymphocyte count >4.65/mm^3^ had a significantly higher probability of CMV disease-free survival than those with counts classified as NI or ≤4.65/mm^3^ *(p <* 0.01) (Figure 4C). The probability of CMV disease-free survival was also higher in R+ KTRs with TEMRA γδ T lymphocytes count > 4.65/mm^3^ than in the group gathering R+ KTRs with TEMRA γδ T lymphocytes count “NI” and ≤4.65/mm^3^ (*p* = 0.02) (Figure 4D).

Univariate analysis identified total lymphocyte counts >761/mm^3^ [HR 0.35 (95% CI: 0.12–0.99); *p* = 0.05], Vδ2^neg^ γδ T lymphocyte counts >7.95/mm^3^ [HR 0.31 (95% CI: 0.11–0.84); *p* = 0.02] and TEMRA γδ T lymphocyte counts >4.65/mm^3^ [HR 0.28 (95% CI: 0.09–0.87); *p* = 0.03] as factors associated with CMV disease (Table 6). In the multivariate analysis of R+ KTRs, only a TEMRA γδ T lymphocyte count >4.65/mm^3^ remained independently associated with protection against CMV disease [HR 0.27 (95% CI: 0.09–0.85); *p* = 0.03] (Table 7).

**TABLE 6:** Univariate analysis of CMV disease risk factors in R+ patients.

Variables	HR	95% CI	*p* value
Age	1.01	0.97–1.04	0.76
Sex (reference group: male)	1.10	0.40–2.9	0.87
Thymoglobulin	1.63	0.60–4.40	0.34
mTOR inhibitors before CMV disease	0.16	0.11–3.66	>0.99
Steroids	1.08	0.35–3.33	0.89
Rejection before CMV disease	1.00	0.99–1.01	0.16
Lymphocytes count >0.761 G/L	0.35	0.12–0.99	0.05
Vδ2^neg^ γδ T lymphocyte count >7.95/mm^3^	0.31	0.11–0.84	0.02
TEMRA γδ T lymphocyte count >4.65/mm^3^ *versus* ≤4.65/mm^3^ and NI	0.28	0.09–0.87	0.03

HR: hazard ratio.

CI: confidence interval.

mTOR: mammalian target of rapamycin.

R+: Recipient positive for CMV, serology.

NI: not interpretable.

**TABLE 7:** Multivariate analysis of CMV disease risk factors in R+ patients.

Variables	HR	95% CI	*p* value
Rejection before CMV disease	1.32	0.17–9.99	0.79
TEMRA γδ T lymphocyte count >4.65/mm^3^ *versus* ≤4.65/mm^3^ and NI	0.27	0.09–0.85	0.03

HR: hazard ratio.

CI: confidence interval.

NI: not interpretable.

## Discussion

In this retrospective, single-center cohort study, KTRs with a TEMRA γδ T lymphocyte count greater than 4.65/mm^3^ at the end of antiviral prophylaxis showed a significantly lower incidence of post-prophylaxis CMV disease during the first 2 years after transplantation. In the overall population, including both D+R- and R+ KTRs, this biomarker did not perform better than CMV serostatus in predicting the occurrence of CMV disease. However, it was independently associated with protection against CMV disease in the R+ population, demonstrating a predictive ability of 97.7% for CMV protection in this subgroup.

The usefulness of several immunomonitoring assays/biomarkers after prophylaxis withdrawal has been studied, but most of them were focused on the CD8^+^ αß T lymphocytes. In 2009, Kumar et al. assessed both D+R- and R+ KTRs, showing that a positive QuantiFERON-CMV assay at the end of prophylaxis was associated with a decreased risk of CMV disease during the first 6 months post-transplantation [2/38 (5.3%) versus 16/70 (22.9%), *p* = 0.038] [9]. In this study, 32 (29.6%) KTRs had indeterminate QuantiFERON-CMV results and were classified as negative. Following this initial study, Manuel et al. conducted a multicenter prospective study in 2013 focused on D+R- KTRs. In this study, QuantiFERON-CMV was performed at the end of prophylaxis, and KTRs were followed for 1 year. Among 127 KTRs, 31 (25%) had a positive QuantiFERON-CMV result, 81 (65.3%) were negative, and 12 (9.7%) had indeterminate results. During the first post-transplant year, KTRs with a positive result had a lower incidence of CMV disease than those with a negative or indeterminate result (6.4%, 22.2%, and 58%, respectively; *p* < 0.001). The assay had a high positive predictive value (93%) but a low negative predictive value (24%) [10]. More recently, Fernandez-Ruiz et al. assessed the post-prophylaxis QuantiFERON-CMV test in R+ KTRs receiving anti-thymocyte globulins. They found no significant difference in the incidence of CMV infection between QuantiFERON-CMV positive and negative groups during the first-year post-transplant (45.8% versus 36.1%; *p* = 0.244). The discrepancy with the study of Manuel et al. could be explained by differing endpoints: Fernandez-Ruiz et al. focused on CMV infection, while Manuel et al. focused on CMV disease [27].

Jarque et al. focused on the association of a positive ELISpot at the end of prophylaxis and the incidence of CMV disease during the first-year post-transplantation in R+ KTRs. They found significantly lower IFN-γ–producing T-cell frequencies against both IE-1 and pp65 CMV antigens in KTRs who later developed CMV infection. IE-1 cell-mediated immunity (CMI) was the strongest predictor of protection against late-onset CMV infection, with a positive predictive value of 90.8% [28]. Finally, Kumar et al. published a multicenter prospective study focusing on the predictive value of ELISpot at the end of prophylaxis in R+ and D+R- KTRs, finding a significantly lower incidence of CMV events in ELISpot positive R+ KTRs, with a positive predictive value above 97% [29].

*In vitro*, γδ T cells inhibit replication and kill infected cells [20], a protective role supported by animal studies [21]. Their expansion in peripheral blood parallels that of CD8^+^ T cells following infection [25] and 8 weeks after treatment initiation, γδ T cell expansion is associated with the absence of CMV recurrence [19].

In this study, we tried to analyze the ability of γδ T cells to predict CMV disease at the end of prophylaxis. We found similar predictive performance for TEMRA γδ T lymphocyte counts above 4.65/mm^3^ than ELISPOT at the end of prophylaxis in R+ KTRs, with a sensitivity of 52.2%, specificity of 82.3%, positive predictive value of 97.7%, and negative predictive value of 10.7%. The high positive predictive value reflects a low incidence of CMV disease in KTRs with TEMRA γδ T lymphocyte counts higher than 4.65/mm^3^ within the first 2 years post-transplantation. This biomarker of the anti-CMV immune response could then complement the ELISPOT or QuantiFERON assays in order to better predict CMV disease and better guide the prevention strategy. It would be particularly interesting to analyze TEMRA lymphocytes levels in patients who do not develop disease despite lacking CD4+/CD8+ T-cell responses or those who develop disease despite having these responses.

TEMRA γδ T lymphocytes appear to be a promising biomarker for the development of a γδ T lymphocyte-mediated adaptive response. However, it has been shown that TEMRA cells can display significant heterogeneity, with dysfunctional phenotypes (PD-1+, CD85j+) linked to an increased risk of CMV infections [30]. Further research is needed to refine the predictive value of TEMRA γδ T lymphocyte counts by incorporating the functional status of these cells. Notably, the functionality of these cells seems to improve in KTRs maintained on mTOR inhibitors compared to those on mycophenolate-based treatments, which may explain the lower CMV disease incidence associated with mTOR inhibitors in our study.

Our study has some limitations. Its retrospective, single-center design underscores the need for confirmation in prospective studies. The second limit is that this technique is not currently standardized. The third limitation is the large proportion of patients with non-informative (NI) results [160/344 (46.5%)]. Similar to the QuantiFERON-CMV assay, this result may be indicative of a weak CMV immune response, as these patients had lower total lymphocytes count, lower Vδ2^neg^ γδ T lymphocyte counts and exhibited more CMV disease than those with TEMRA γδ T lymphocyte counts above 4.65/mm^3^. Since these non-significant findings are attributable to the insufficient number of circulating γδ T cells, resolving this issue may require increasing the number of cells analyzed through flow cytometry to improve sensitivity. Additionally, the main findings of our study apply to R+ KTRs, who are not the highest-risk group for CMV disease.

Future interventional studies are needed to determine whether TEMRA γδ T lymphocyte counts can improve CMV immune risk stratification and guide personalized CMV prevention strategies. Currently, the QuantiFERON-CMV and ELISpot-CMV assays can be used: at 4–6 weeks post-transplantation in R+ KTRs receiving thymoglobulin and universal prophylaxis to discontinue antivirals early in those with positive results [16], 2/at 2 weeks post-transplantation in R+ KTRs managed with a preemptive approach to stop PCR monitoring in those with a positive result [18]. Adding TEMRA γδ T lymphocyte counts to the arsenal of CMV cell-mediated immunity assays could enhance immune-guided CMV prevention, particularly in R+ KTRs with negative QuantiFERON-CMV or ELISpot-CMV results.

## Data Availability

The raw data supporting the conclusions of this article will be made available by the authors, without undue reservation.
