# Supplementary figures and images for: Effector-Memory γδ T Lymphocytes Predict CMV Disease After the Withdrawal of Prophylaxis in Kidney Transplant Recipients
Source: Transpl Int. 2025 Jul 16;38:14339. doi: 10.3389/ti.2025.14339 (PMC12307245; doi:10.3389/ti.2025.14339)

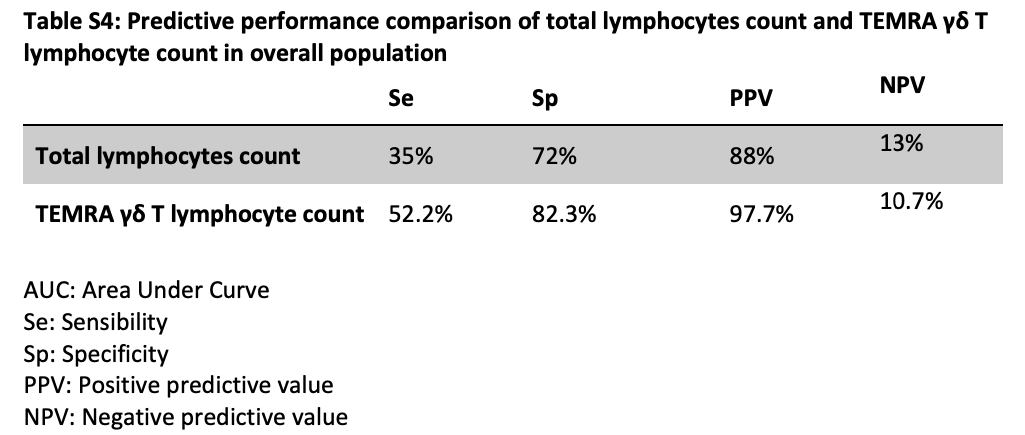

Supplement: Supplementary file 1 [file Image5.png]

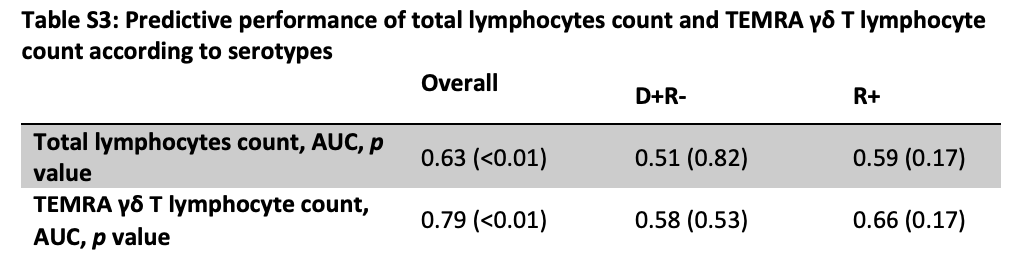

Supplement: Supplementary file 2 [file Image4.png]

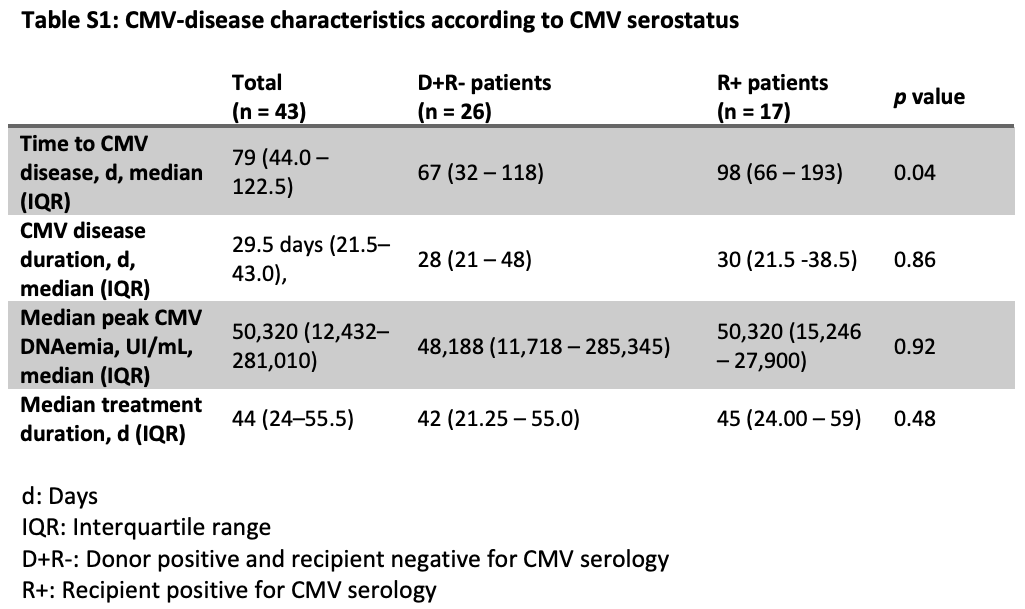

Supplement: Supplementary file 3 [file Image2.png]

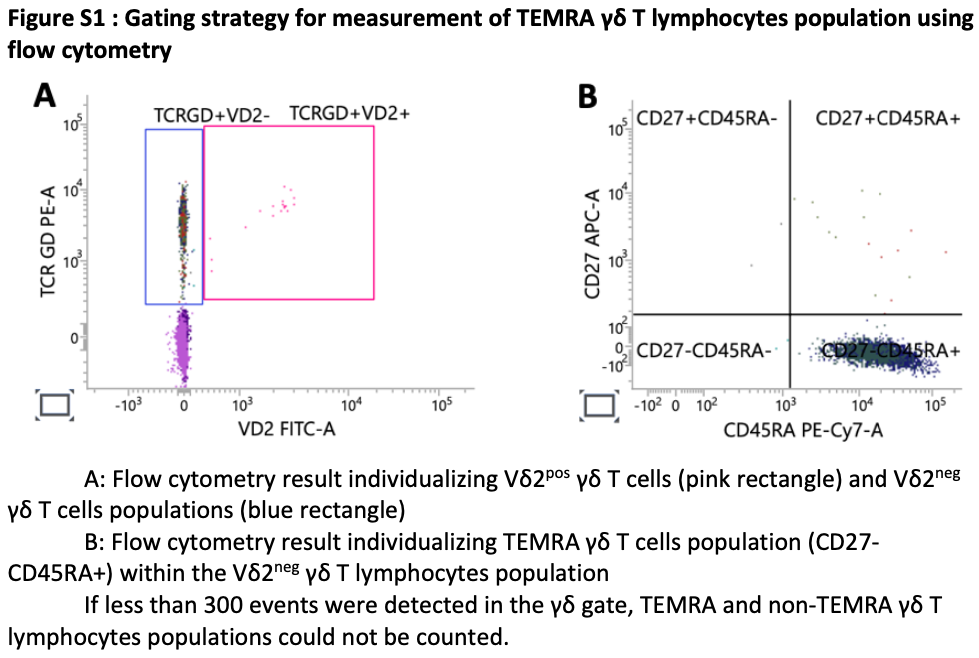

Supplement: Supplementary file 4 [file Image1.png]

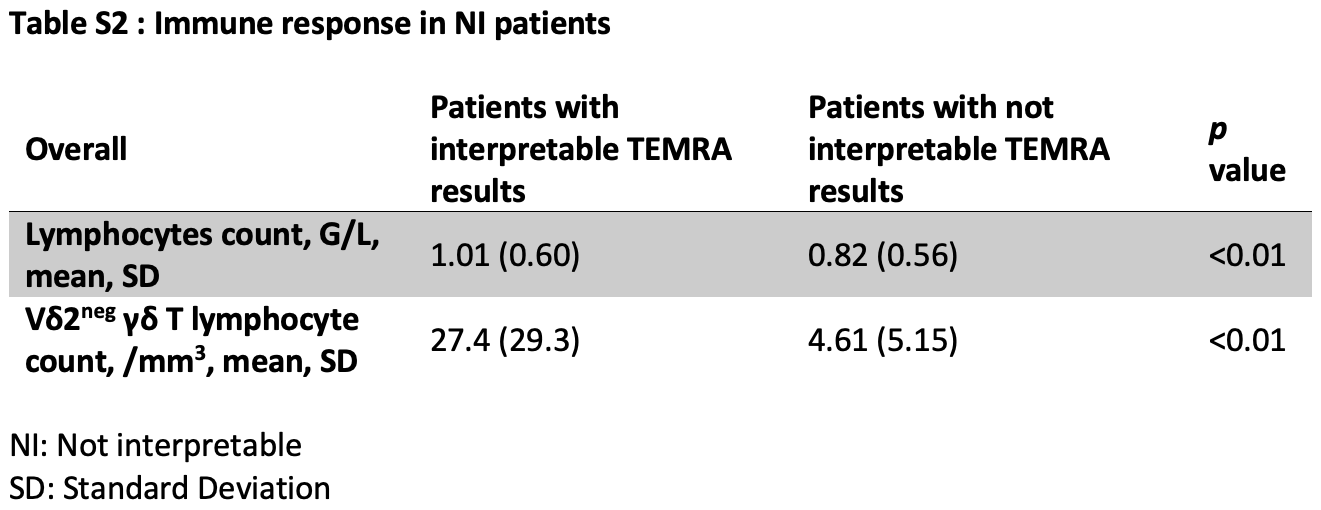

Supplement: Supplementary file 5 [file Image3.png]
